# Dr. Alexander J. C. Skeene

**Published:** 1900-08

**Authors:** 


### OBITUARY.

Dr. Alexander J. C. Skeene, of Brooklyn, died at his summer home in the Catskill Mountains, July 4, 1900, aged 62 years. He was born of noted Scotch parentage at Fyrie, Aberdeenshire, Scotland, and came to this country in 1857. Soon afterward he entered the University of Michigan, but took his doctorate degree at Long Island College Hospital in 1863. He served as a medical officer in the civil war and is credited with the organisation of a hospital corps that is yet in vogue both in the U. S. Army and the State National Guard.

Dr. Skeene returned to Brooklyn after leaving the military service and became a teacher of medicine in his Alma Mater, and later was appointed professor of gynecology in the same institution, retiring from active relations in the faculty of the Long Island Medical College about a year ago. He was a writer of distinction, did much original work in his special field, was a lover and patron of art, a sculptor of no mean ability, and was altogether a useful citizen and lovable man. He is survived by a widow and an adopted daughter.

The New York *Tribune* pays editorially in its issue of July 7th, a beautiful tribute to his memory, which we reproduce as follows:

A LOSS TO HUMANITY.

In the death of Dr. A. J. C. Skeene, of Brooklyn, the world sustains a grievous loss. The community loses an ideal citizen, a man who, with rare unselfishness and singleness of purpose, labored to lessen pain and suffering and to increase health and life—of whom, indeed, it might without irreverence be said that he lived and labored that others might have life and might have it more abundantly. Beginning his career with the creation of the now invaluable army ambulance service, he has closed it with the foundation of what was designed to be one of the noblest and most beneficent of charities. The profession which he adorned suffers a loss which is to be estimated by members of that profession more justly than by laymen. The world knows, however, of his rare judgment in diagnosis, his marvelous ability in surgical operation, and his authority as a teacher and writer, recognised wherever the English language and, indeed, most other civilised languages are used.

Most keen of all, perhaps, is the sense of personal loss which comes to innumerable former patients and to friends. His cheery humor, his spontaneous sympathy, his almost more than human tenderness and his unfailing and inspiring hopefulness must remain precious memories to all who have been ministered to by him. To more than a few, who have long leaned upon his strength and who have partaken of his generous bounty, it will now seem as if, in the words of Carlyle's sorrow, the light of their lives has gone out. For such a loss to humanity there could be no consolation were it not for the assurance that greater still has been the world's gain through the activities and achievements of his pure and exalted life.

